# Reactivation of Cytomegalovirus in a Patient with Stevens-Johnson Syndrome-Toxic Epidermal Necrolysis

**Published:** 2013-06

**Authors:** Mohamed Rida Tagajdid, Taoufik Doblali, Hicham Elannaz, Salaheddine Hammi, Bouchra Belfequih, Saâd Mrani

**Affiliations:** 1 Laboratory of Virology, Mohamed V Souissi University, Mohamed V Military Teaching Hospital, Rabat, BP, Morocco;; 2 Department of Internal Medicine, Mohamed V Military Teaching Hospital, Rabat, BP, Morocco

**Keywords:** Stevens-Johnson syndrome, Toxic epidermal necrolysis, Cytomegalovirus, Pneumonia

## Abstract

Stevens-Johnson syndrome (SJS) and toxic epidermal necrolysis (TEN) are severe adverse cutaneous reactions to drugs. We describe the case of a 19 year old patient with SJS/TEN overlap syndrome, who developed severe interstitial pneumonia after she had received antiepileptic drugs. A cytomegalovirus infection was diagnosed by Real Time Polymerase Chain Reaction (RT-PCR) detection on Bronchoalveolar lavage. Based on observations on biological data, temporal relationship, and clinical features, it could be inferred that the reactivation of cytomegalovirus with viral replication can predispose a person to TEN-SJS. We discuss here, in the light of the current literature, the probable association between drug-induced SJS-TEN and fulminant reactivation of cytomegalovirus.

## Introduction

Stevens-Johnson syndrome (SJS) and toxic epidermal necrolysis (TEN) are variants of acute, rapidly progressive mucocutaneous reactions. These reactions differ only in their body surface area (BSA) involvement: whereas SJS is the less severe condition insofar as skin sloughing is limited to less than 10% of the BSA, TEN involves sloughing of more than 30% of the BSA. The SJS/TEN overlap syndrome describes patients with the involvement of greater than 10%, but less than 30% of the BSA.^1^ TEN and SJS (TEN-SJS) is a life-threatening condition, where there is extensive detachment of the skin characterized by full-thickness necrosis of the epidermis. Most cases of SJS-TEN are drug-induced. In patients with no drug use, TEN-SJS is induced by chemicals, *Mycoplasma pneumonia,* immunization, and viral infections.^2^^,^^3^

We describe here a patient with the SJS/TEN overlap syndrome, who developed severe interstitial pneumonia caused by a cytomegalovirus in the wake of treatment with antiepileptic drugs. 

## Case History

Miss. N, a 19 year old Moroccan woman, without a history of drug or food allergy, was prescribed Phenobarbital (100 mg/day) and Valproate (1000 mg/day) for epilepsy one month prior to her latest hospital admission due to erythroderma, fever (39.8°C), and dyspnea. 

On admission, the patient was conscious and reported chills, coughs, and rhinorrhea of two days’ duration. She had developed rash on her face six days after starting epilepsy treatment, with the condition progressing rapidly to her trunk, limbs, neck, and chest over the next four days. The facial rash subsequently evolved into pustules. 

On examination, there were multiple erosions over the mouth and vulva, and the conjunctiva was inflamed. Nikolsky’s sign was positive. The total extent of the erythematous rash was 55% of the BSA. Histological investigation of the injured skin revealed an aspect for drug eruption (extensive epidermal necrosis, focal subepidermal necrotic blisters, melanin incontinence, and moderate perivascular lymphocytic infiltrate in the absence of eosinophils, neutrophils, and viral inclusions). Immunofluorescence markers showed negative staining. The diagnosis of SJS-TEN caused by antiepileptic drugs was established. 

On the other hand, laboratory testing revealed anemia, eosinophilia, increased inflammatory markers, and white blood cell count of 10×10^9^ /µL. Chest X-ray demonstrated multifocal patch consolidations with ground-glass opacity in both lungs. No bacterial pathogen was isolated in the respiratory tract, urine, and blood. Viral serology (HIV and hepatitis B and C) was negative. Real Time Polymerase Chain Reaction (RT-PCR) (R-gène® Kits) of bronchoalveolar lavage (BAL) revealed that the cause of the respiratory symptom was cytomegalovirus (CMV); the finding was thereafter confirmed by cell culture on MRC-5 cells. Prior serology data showed that our patient had already sustained a primary CMV infection at 6 years old, which was in favour of the current reactivation. As was expected, RT-PCR in blood showed fulminant viremia with 459 copies/ml (threshold: 350). 

The antiepileptic drugs were withdrawn and anticoagulant therapy, parenteral analgesia, eye drops, and mouth antiseptic therapy were initiated. Intravenous Ganciclovir (10 mg/kg/day) was started. Oxygen therapy was initiated with parenteral nutrition and adequate hydration. Serial BAL assays were negative six days after the commencement of antiviral therapy, and the skin lesions began to heal without the need to introduce corticosteroids. The patient’s condition improved, and she was transferred to the general ward with a prescription of Ganciclovir for a further two weeks.

## Discussion

TEN and SJS are severe adverse cutaneous drug reactions.^4^^,^^5^ Treatment with corticosteroids is recommended but not necessarily effective.^5^^-^^7^ Several drugs are at “high” risk of inducing TEN-SJS.^1^ *Mycoplasma pneumoniae* and *herpesvirus* infections are well-documented causes. In some rare cases, however, the etiology remains unknown.^8^

The case presented here suggests a link between CMV replication and drug-induced TEN-SJS. Our patient developed TEN-SJS and in parallel, pneumonia caused by CMV reactivation, six days after the initiation of antiepileptic drugs. It is deserving of note that there are also cases in which the viral reactivation is accompanied by flu-like symptoms; therefore, it is possible that the six-day period before admission represents the duration of reactivation in our patient. T*emporal relationship and clinical features* strongly suggest CMV with viral replication, predisposing the patient to TEN-SJS. It is clear that without a thorough understanding of the underlying mechanisms involved, it is difficult to establish a direct causal link between CMV and drug hypersensitivity. However, a relationship between viral infections and the simultaneous or subsequent development of drug-induced rash has been observed in a number of clinical situations, while the full cascade of events leading from viral infections to the development of drug allergy in humans remains poorly understood. Ampicillin rash during infectious mononucleosis and an increased risk for developing drug-induced rash in AIDS are well-known examples of this relationship.^3^ The herpesvirus family is the most likely candidate to be able to greatly influence immune responses because *herpesviruses* can induce and maintain a potent memory T cell response due to their common properties of ubiquitous prevalence in human populations and the capacity to grow in lymphoid cells.^8^ Specific viral infections have been shown to increase CD95 (Fas) and/or Fas Ligand expression and increase sensitivity to Fas/Fas Ligand-dependent apoptosis.^3^

Treatment strategies for TEN-SJS associated with CMV include treatment of the cause with Ganciclovir, avoidance of possible offending drugs associated with TEN-SJS, and avoidance of systemic steroids assuming that the underlying mechanism is most probably the interaction between CMV and some enzymes that detoxify, such as cytochrome P450. The offending drugs associated with TEN-SJS cause the deposition of the toxic and immunogenic metabolites of these drugs in the epidermis and lead to a series of immune reactions that culminate in TEN-SJS.^3^ The hypothesis generated is whether or not TEN-SJS is linked to fulminant CMV infection and whether or not CMV can trigger an interaction between cytotoxic T-lymphocytes, natural killer cells, and keratinocytes. Further observational studies are warranted. 

## Conclusion

The case presented herein illustrates that a possible CMV interstitial pneumonia (secondary to CMV reactivation) may predispose a patient to SJS-TEN. Implications for clinical practice include the notions that SJS-TEN is a potential adverse effect of some drugs and that patients at risk for the development of TEN-SJS may be identified by measuring CMV loads during the first few days after onset, even if CMV IgM and IgG levels are negative. 
